# Remodeling of t-system and proteins underlying excitation-contraction coupling in aging versus failing human heart

**DOI:** 10.1038/s41514-021-00066-7

**Published:** 2021-05-28

**Authors:** Yankun Lyu, Vipin K. Verma, Younjee Lee, Iosif Taleb, Rachit Badolia, Thirupura S. Shankar, Christos P. Kyriakopoulos, Craig H. Selzman, William Caine, Rami Alharethi, Sutip Navankasattusas, Thomas Seidel, Stavros G. Drakos, Frank B. Sachse

**Affiliations:** 1 Nora Eccles Harrison Cardiovascular Research and Training Institute, University of Utah, Salt Lake City, UT USA; 2 Department of Biomedical Engineering, University of Utah, Salt Lake City, UT USA; 3 Utah Transplantation Affiliated Hospitals (U.T.A.H.) Cardiac Transplant Program, University of Utah Health & School of Medicine, Intermountain Medical Center, VA Medical Center, Salt Lake City, UT USA; 4 Institute of Cellular and Molecular Physiology, Friedrich-Alexander-Universität Erlangen-Nürnberg (FAU), Erlangen-Nürnberg, Germany

**Keywords:** Senescence, Ageing

## Abstract

It is well established that the aging heart progressively remodels towards a senescent phenotype, but alterations of cellular microstructure and their differences to chronic heart failure (HF) associated remodeling remain ill-defined. Here, we show that the transverse tubular system (t-system) and proteins underlying excitation-contraction coupling in cardiomyocytes are characteristically remodeled with age. We shed light on mechanisms of this remodeling and identified similarities and differences to chronic HF. Using left ventricular myocardium from donors and HF patients with ages between 19 and 75 years, we established a library of 3D reconstructions of the t-system as well as ryanodine receptor (RyR) and junctophilin 2 (JPH2) clusters. Aging was characterized by t-system alterations and sarcolemmal dissociation of RyR clusters. This remodeling was less pronounced than in HF and accompanied by major alterations of JPH2 arrangement. Our study indicates that targeting sarcolemmal association of JPH2 might ameliorate age-associated deficiencies of heart function.

## Introduction

In 2050, 25% of the population in developed countries is expected to have an age of 60 years and above^1^. Aging is associated with an increased incidence and decreased threshold for the development of cardiovascular diseases, for instance, heart failure (HF) with preserved ejection fraction^2,3^. Aging of healthy hearts is associated with structural remodeling such as left ventricular wall thickening and myocardial fibrosis^4–6^. Also, age-associated functional changes of the myocardium includes prolonged contraction, impaired relaxation, and reduced sensitivity to adrenergic stimuli^4^. While such changes are known to be hallmarks of cardiac diseases, the adaptation of the left ventricular mechanics, allows the heart to preserve function during normal aging to some extent^7^. Thus, important hemodynamic measures, such as stroke volume and left ventricular ejection fraction (LVEF) at rest, change only marginally during aging. However, significant limitations of this adaptation were observed during exercise and in the presence of cardiac diseases^4–6^. Beyond the quantitative measures, aging of the heart reduces the quality of life of older adults^8^.

While it is well established that the aging, healthy heart progressively remodels towards a senescent phenotype at the tissue and organ level^4,9^, age-associated remodeling at the cellular level is not well studied. Beyond compensation of age-associated loss of ventricular cardiomyocytes by hypertrophy^10^, phenotypes of functional changes and structural remodeling, mechanisms for preservation of human heart function, and limitations thereof, are largely unknown. A major gap in the understanding of cardiac aging is the lack of information on the age-associated remodeling of excitation-contraction (EC) coupling in cardiomyocytes. In cardiomyocytes, EC coupling is based on calcium signaling that connects electrical excitation of the sarcolemma to mechanical contraction of sarcomeres^11^. Current understanding of cardiac EC coupling revolves around the couplon^12^, which comprises a cluster of ryanodine receptors (RyRs) in the sarcoplasmic reticulum (SR) and a cluster of voltage-gated L-type calcium channels (LCCs) in the sarcolemma^13^. Proximity of RyR and LCC clusters is required for their functional interaction in the form of calcium-induced calcium release^14^. This proximity is facilitated by junctional complexes that tie the SR membrane to the sarcolemma. Junctophilin-2 (JPH2) is thought to be a major protein of these junctional complexes in cardiomyocytes^15,16^. Comparison of JPH2 knockdown to wild-type animals revealed a reduction of junctional complexes and decreased gain of EC coupling^16^. In contrast, increased expression of JPH2 caused enlargement of junctional complexes, did not increase baseline functions, and attenuated HF development^17,18^. Various mutations and down-regulation of JPH2 have been linked to cardiac diseases such as HF, hypertrophy, and arrhythmia^19–21^.

In ventricular cardiomyocytes, couplons are commonly associated with a system of sarcolemmal invaginations, the transverse tubular system (t-system)^22–24^. The t-system rapidly carries electrical excitation into the myocyte interior, which synchronizes activation of couplons, and thus calcium release, within the cell. Ventricular cardiomyocytes from mammals, including humans, present a dense t-system, and its integrity is crucial for efficient EC coupling. Several cardiac diseases including HF are associated with loss of t-system and alterations of its phenotype^25^. We and others showed that loss of t-system and the associated loss of couplons in ventricular myocytes from HF patients lead to spatiotemporal heterogeneity of cytosolic calcium transients^26^, which is linked to deleterious alterations of the contractile function^27,28^. Also, the integrity of the t-system was found to be crucial for cardiac recovery in HF patients undergoing left ventricular assist device (LVAD) therapy^26^. Restoration of the t-system is thought to be directly implicated in the myocardial functional recovery after cardiac resynchronization therapy^29^.

Investigations of age-associated remodeling of EC coupling-related structures have not been performed in humans and only sparsely in animal models of aging. However, functional studies on animal models demonstrated multi-faceted, age-associated changes of electrophysiology, calcium homeostasis, EC coupling, and contractility of cardiomyocytes^30,31^. Aging was found to attenuate the effects of β-adrenergic stimulation on EC coupling^32^ and contractility^33^. Animal models employed were primarily rats, mice, and rabbits. Functional changes were species-dependent, which complicates the translation of findings to the understanding of human aging. Also, remodeling differed for sex. For instance, peak cytosolic calcium, SR calcium uptake, and contractility were attenuated in cardiomyocytes of aging male but not female rodents^34,35^. Functional studies on aging effects on the EC coupling in the human heart are sparse. A recent study on atrial myocytes from patients undergoing cardiac surgery suggested that aging causes major deficiencies in calcium homeostasis^36^, but due to the presence of heart disease in these patients, it is unclear how these findings relate to the aging of the healthy heart.

## Results

Here, we describe age-associated remodeling of structures and proteins underlying EC coupling in human ventricular myocytes, using confocal microscopy and 3D reconstruction. We examined cardiac tissue samples from donors of a wide range of ages. The hearts were not used for transplantation due to non-cardiac reasons, e.g. size issues and infections. We tested the hypothesis that reduced JPH2 cluster density causes remodeling of the t-system and dissociation of RyRs from the sarcolemma. We investigated mechanisms of reduced JPH2 cluster density using quantitative polymerase chain reaction (qPCR) and western blotting. To shed light on the causes of increased incidence of HF in the aging population, we compared age-associated remodeling to remodeling in advanced chronic HF patients. Finally, we explored if the accumulation of lipofuscin, an established marker of cardiomyocyte aging^37^, predicts microstructural remodeling in our donor and HF patient population.

### Study population and clinical measurements

**Fig. 1:**
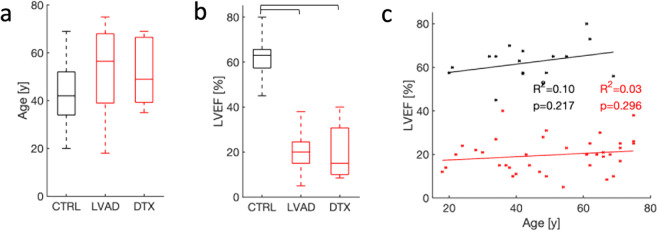
Age and LVEF for donors and HF patients. Boxplot of **a** age and **b** LVEF of donors and patients undergoing LVAD implantation and DTX. Age differences in donors and patients were not significant. LVEF of donors was in the normal range, but markedly reduced in LVAD and DTX patients. **c** Age-LVEF relationships of donors and patients. The effect of age on LVEF was not significant in donors and patients.

The study population comprised 17 donors, 42 advanced chronic HF patients who underwent LVAD implantation, and 7 advanced chronic HF patients who underwent direct heart transplant (DTX). Demographic and clinical information on donors and HF patients is provided in Tables 1 and 2, respectively. Commonly, donors and HF patients were of the female and male sex, respectively. This distribution reflects the higher availability of hearts from female donors and the higher prevalence of male HF patients. Based on hemoglobin A1c (HbA1c) levels, donors were non-diabetic. LVAD patients exhibited widely varying HbA1c levels. The donors had a body mass index (BMI) close to the mean BMI of the US population in 2015–16 (29.1 kg/m^2^)^38^. The age of donors, LVAD, and DTX patients ranged from 20 to 69, 19 to 75, and 35 to 69 years, respectively (Fig. 1a).

**Table 1:** Baseline demographic and clinical characteristics of donor population.

Age (years)	44.0 ± 3.3
Male, *n* (%)	3.0 (17.6%)
Height (cm)	166.0 ± 1.7
Weight (kg)	72.0 ± 3.6
BMI (kg/m^2^)	26.1 ± 5.5
HbA1c (%)	5.3 ± 0.4
LVEF (%)	63.2 ± 1.7
Left ventricular end-diastolic diameter (cm)	4.0 ± 0.1

Values are shown in *n* (%) or mean ± standard error when appropriate.

*BMI* body mass index, *HbA1c* hemoglobin A1c.

**Table 2:** Baseline demographic and clinical characteristics of HF population.

	LVAD	DTX	HF
Age (years)	52.1 ± 2.7	48.2 ± 5.3	50.1 ± 4.0
Male, *n* (%)	34 (80.9)	5 (71.4)	39 (76.1)
Ischemic cardiomyopathy, *n* (%)	17 (40.4)	N/A	17 (40.4)
Non-ischemic cardiomyopathy, *n* (%)	24 (57.1)	N/A	24 (57.1)
NYHA class III, *n* (%)	10 (23.8)	1 (14.2)	11 (19.0)
NYHA class IV, *n* (%)	31 (73.8)	6 (85.7)	37 (66.2)
Duration of heart failure (years)	6.3 ± 1.2	N/A	6.3 ± 1.2
LVEF (%)	19.9 ± 1.1	20.6 ± 4.8	20.2 ± 2.9
Left ventricular end-diastolic diameter (cm)	6.5 ± 0.1	6.4 ± 0.5	6.4 ± 0.3
HbA1c (%)	6.2 ± 0.9	N/A	6.2 ± 0.8
Brain natriuretic peptide (pg/ml)	1343.5 ± 169.2	1285.1 ± 427.2	1314.3 ± 298.2
Creatinine (mg/dl)	1.33 ± 0.07	1.73 ± 0.05	1.5 ± 0.6
Sodium (mmol/l)	134.2 ± 0.7	134.5 ± 0.7	134.3 ± 0.7
Hemoglobin (g/dl)	12.7 ± 0.3	11.2 ± 1.0	11.9 ± 0.6
Systolic arterial pressure (mm Hg)	103.1 ± 2.4	111.1 ± 6.4	107.1 ± 4.4
Diastolic arterial pressure (mm Hg)	64.9 ± 2.4	67.8 ± 2.4	66.3 ± 2.4
Mean right atrial (mm Hg)	12.3 ± 1.0	8.8 ± 2.0	10.5 ± 1.5
Pulmonary capillary wedge pressure (mm Hg)	25.2 ± 1.5	20.0 ± 3.0	22.6 ± 2.2
Systolic pulmonary arterial pressure (mm Hg)	54 ± 2.1	37.5 ± 3.2	45.8 ± 2.6
Diastolic pulmonary arterial pressure (mm Hg)	26.7 ± 1.5	22.1 ± 1.4	24.4 ± 1.4
Pulmonary vascular resistance (Wood units)	3.8 ± 0.3	1.4 ± 0.2	2.6 ± 0.3
Cardiac index (l/min/m^2^)	1.8 ± 0.07	2.1 ± 0.1	1.9 ± 0.08

Values are shown in *n* (%) or mean ± standard error when appropriate.

*NYHA* New York Heart Association, *HbA1c* hemoglobin A1c.

Heart function in the study population was assessed from LVEF measured via echocardiography. LVEF ranged from 45 to 80, 5 to 38, and 9 to 40% in donors, LVAD, and DTX patients (Fig. 1b). Mean LVEF was higher in donors than in LVAD and DTX patients, reflecting functional deficiencies of diseased hearts. We pooled data from LVAD and DTX patients, as both were suffering from similar advanced chronic HF, for subsequent analyses of tissue microstructure. This is a standard approach in HF studies that include LVAD and DTX patients. Effects of age on LVEF in donors and HF patients were not significant (Fig. 1c). The range of LVEF in donors and the absence of an age effect on LVEF are in agreement with prior work measuring resting LVEF in subjects without cardiovascular disease and age ranging from 20 to 95 years^39^.

### Image library to investigate age and HF-associated remodeling

We collected samples of the left ventricular myocardium from the donors and HF patients, and imaged the samples at sub-micrometer resolution using scanning confocal microscopy. We generated a library comprising image stacks and 3D reconstructions of 4′,6-diamidino-2-phenylindole (DAPI), wheat germ agglutinin (WGA), and RyR signals in 194 tissue regions. DAPI signal served as a marker for nuclei of myocytes and other cells. WGA marked the extracellular matrix and sarcolemma including the t-system. In addition, the library contained JPH2 image stacks from 150 tissue regions. We extended the library with DAPI, WGA, and RyR images from 34 tissue regions from our prior work^26^. On average, three regions were imaged per donor or patient. Voxel size of the image stacks and 3D reconstructions was 0.1 µm × 0.1 µm × 0.1 µm.

**Fig. 2:**
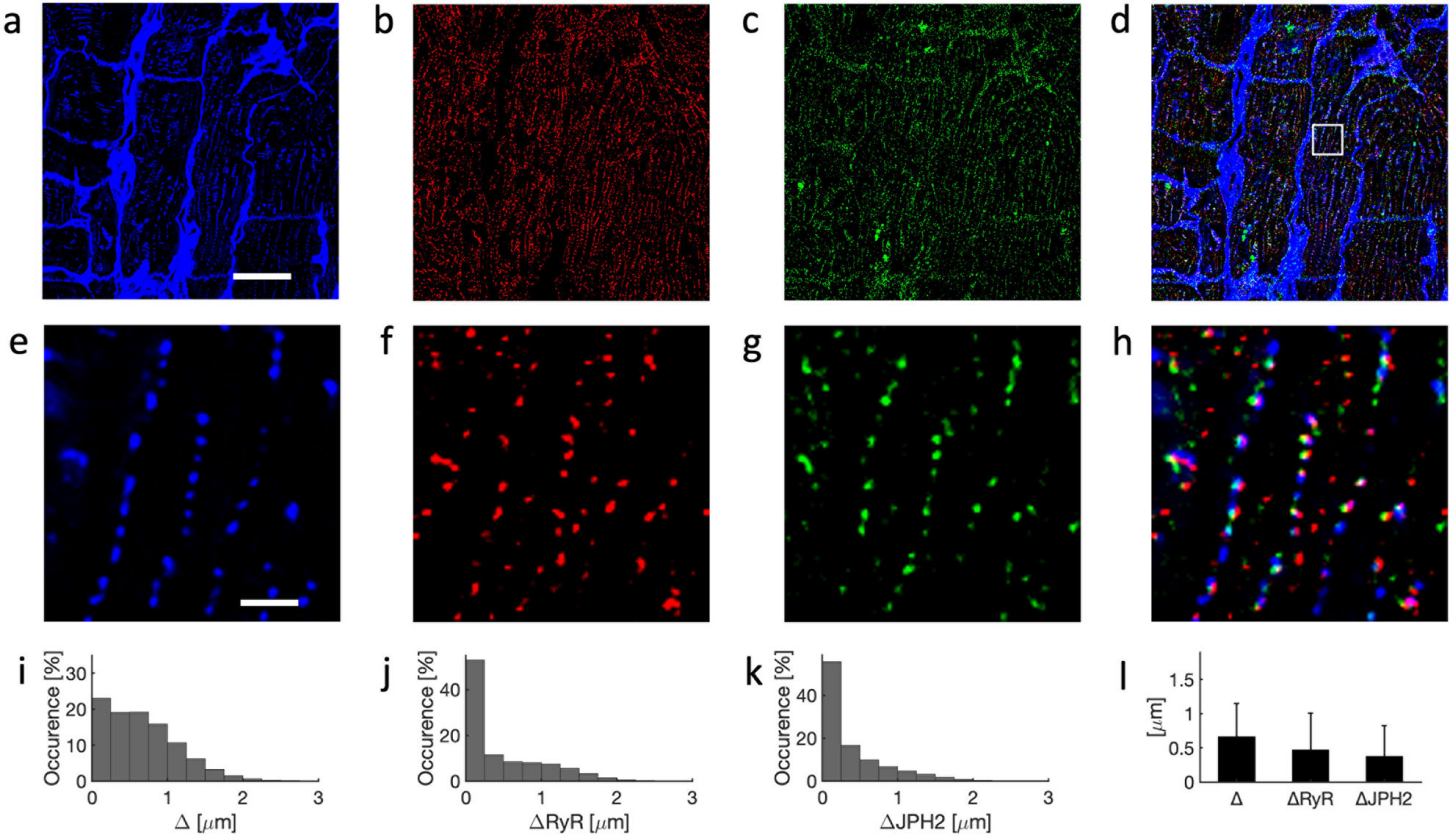
Confocal microscopy of left ventricular myocardium from 21-year-old donor. Image stacks were deconvolved to account for anisotropic blurring and corrected for depth-dependent signal attenuation. Example cross-sectional images from 3D stack for **a** WGA, **b** anti-RyR, and **c** anti-JPH2 antibody labeling. WGA served as a marker of the extracellular spaces and sarcolemma including t-system. **d** Overlay of **a**–**c**. **e**–**h** Zoom-ins of region in **a**–**d** marked with box in **d**. WGA-associated signals describe cross-sections of t-tubules. RyR- and JPH2-associated signals were apparent in small regions reflecting the clustering of these proteins. Histograms of sarcolemmal distances to **i** intracellular sites, **j** RyR clusters, and **k** JPH2 clusters. **l** Bar graph of sarcolemmal distances with error bars identifying their standard deviation. Scale bar in **a** marks 20 µm and applies to **b**–**d**. Scale bar in **e** marks 2 µm and applies to **f**–**h**.

Example images of left ventricular tissue of a 21-year-old donor are shown in Fig. 2. They were acquired by 3D confocal microscopy and processed to account for imaging artifacts. Imaging covered an area of 102.4 µm × 102.4 µm and a depth of 26.5 µm. The imaging yielded information on the microstructure of cardiomyocytes within the extracellular matrix (Fig. 2a) as well as dense intracellular clustering of RyR (Fig. 2b) and JPH2 (Fig. 2c). The magnified images (Fig. 2e–h) present a dense t-system with adjacent RyR clusters. JPH2 signal was found primarily in clusters, which were located in close proximity to t-system and RyR clusters. JPH2 and RyR clusters exhibited a similar size, close to the size of point-spread functions of the confocal microscope. We visualized the 3D reconstruction of t-system, RyR, and JPH2 in Movie 1. Histograms of the sarcolemmal distances of intracellular sites, RyR, and JPH2 clusters are shown in Fig. 2i–k, respectively. The majority of RyR and JPH2 clusters were localized within 0.25 µm of the sarcolemma, which is close to the resolution limit of confocal microscopy and thus indicates that clusters are within couplons. Mean and standard deviations of the distances confirm the sarcolemmal proximity of RyR and JPH2 clusters (Fig. 2l).

**Fig. 3:**
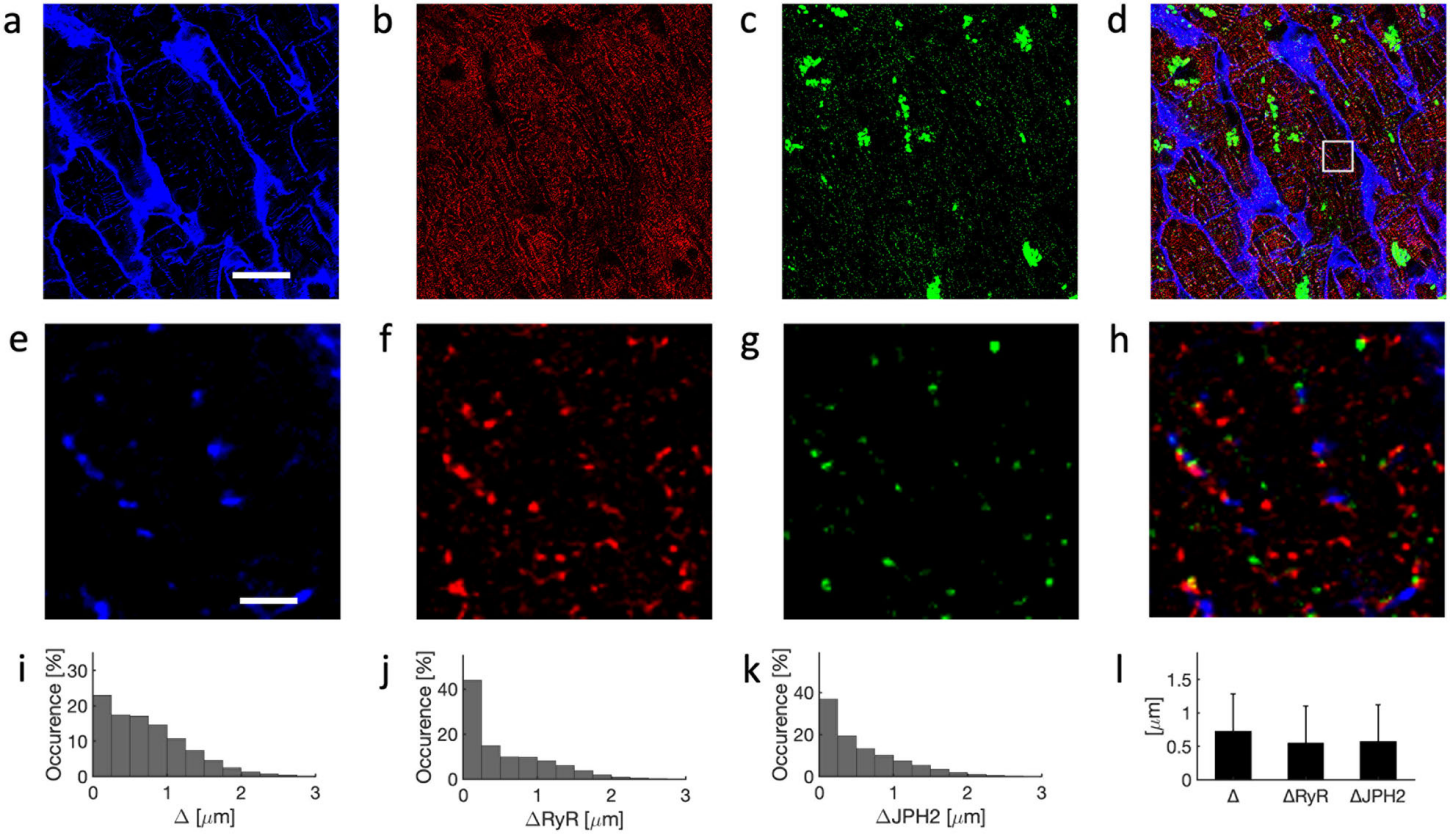
Confocal microscopy of tissue from 69-year-old donor. Example images for labeling with **a** WGA, **b** anti-RyR, and **c** anti-JPH2 antibodies. JPH2 signal was present in small clusters and also in large perinuclear regions. **d** Overlay of **a**–**c**. **e**–**h** Zoom-ins of region in **a**–**d** marked with box in **d**. Histograms of sarcolemmal distances to **i** intracellular sites, **j** RyR clusters, and **k** JPH2 clusters. **l** Bar graph of distances with error bars identifying their standard deviations. Scale bar in **a** marks 20 µm and applies to **b**–**d**. Scale bar in **e** marks 2 µm and applies to **f**–**h**.

Example images from confocal microscopy of left ventricular tissue from a 69-year-old donor are shown in Fig. 3. Similar to observations in the images from the young donor, RyR and JPH2 signals were clustered within the myocytes (Fig. 3f, g). The t-system arrangement appeared less regular and dense vs. the t-system in the young donor (Figs. 3e vs. 2e). Both, RyR and JPH2 clusters were commonly found distal from the t-system. Furthermore, JPH2 clusters appeared less dense than in the young donor heart (Fig. 3h).

In response to illumination with the 633-nm laser used for imaging of JPH2, the images presented large fluorescent regions within cardiomyocytes, which were often close to the nuclei (Fig. 3c, d). Prior work established these autofluorescent regions as lipofuscin, i.e. lipid-containing granules. Lipofuscin is a marker of chronological aging in various cells including cardiomyocytes^37^ and neurons^40^. Visualization with DAPI revealed localization of the autofluorescent regions in perinuclear regions (Supplementary Fig. 1). We confirmed the autofluorescence by applying our imaging protocol to the same samples without fluorescent labeling.

A 3D reconstruction of the t-system, RyR, and JPH2 clusters is shown in Movie 2. Histograms of sarcolemmal distances of intracellular regions showed a similar distribution as in the young donor (Figs. 3i vs. 2i). However, histograms of sarcolemmal distances of RyR and JPH2 clusters presented a smaller degree of sarcolemmal association vs. the young donor (Figs. 3j, k vs. 2j, k, respectively). Information on the distances is summarized in Fig. 3l.

**Fig. 4:**
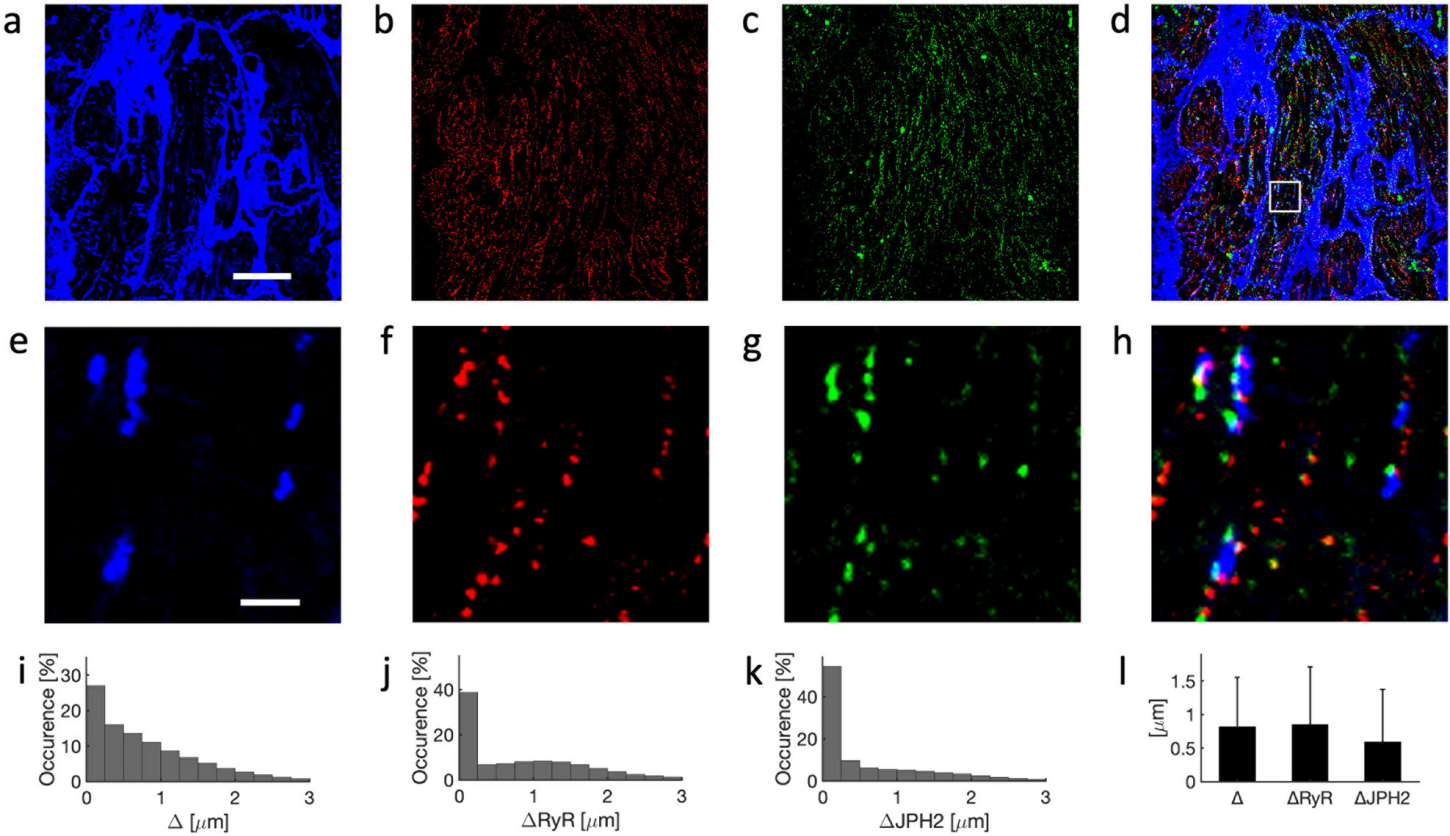
Confocal microscopy of tissue from 19-year-old patient undergoing LVAD implantation. Example images for labeling with **a** WGA, **b** anti-RyR, and **c** anti-JPH2 antibodies. **d** Overlay of **a**–**c**. **e**–**h** Zoom-ins of region in **a**–**d** marked with box in **d**. Histograms of sarcolemmal distances to **i** intracellular sites, **j** RyR clusters, and **k** JPH2 clusters. **l** Bar graph of distances with error bars identifying their standard deviations. Scale bar in **a** marks 20 µm and applies to **b**–**d**. Scale bar in **e** marks 2 µm and applies to **f**–**h**.

We present example images from a 19-year-old patient who underwent LVAD implantation in Fig. 4. The images reflect major remodeling of the t-system characteristic for chronic HF (Fig. 4a, e). Beyond de-tubulation, the images present sheet-like remodeling, which we previously identified as a hallmark of advanced chronic HF^26^. RyR and JPH2 clusters were commonly not associated with the t-system (Fig. 4f, g). Perinuclear accumulations of lipofuscin signal, as demonstrated in the images from the old donor, were not present (Supplementary Fig. 1). The sparse t-system and dissociation of RyR and JPH2 clusters are emphasized in Movie 3. De-tubulation was reflected in pronounced occurrences of intracellular regions remote (>1.5 µm) to sarcolemma including t-system (Fig. 4i). Similarly, histograms of sarcolemmal distances of RyR and JPH2 clusters presented pronounced occurrences of larger distances (Fig. 4j, k).

### Age-associated heterogeneity of t-system and sarcolemmal dissociation of RyR clusters

**Fig. 5:**
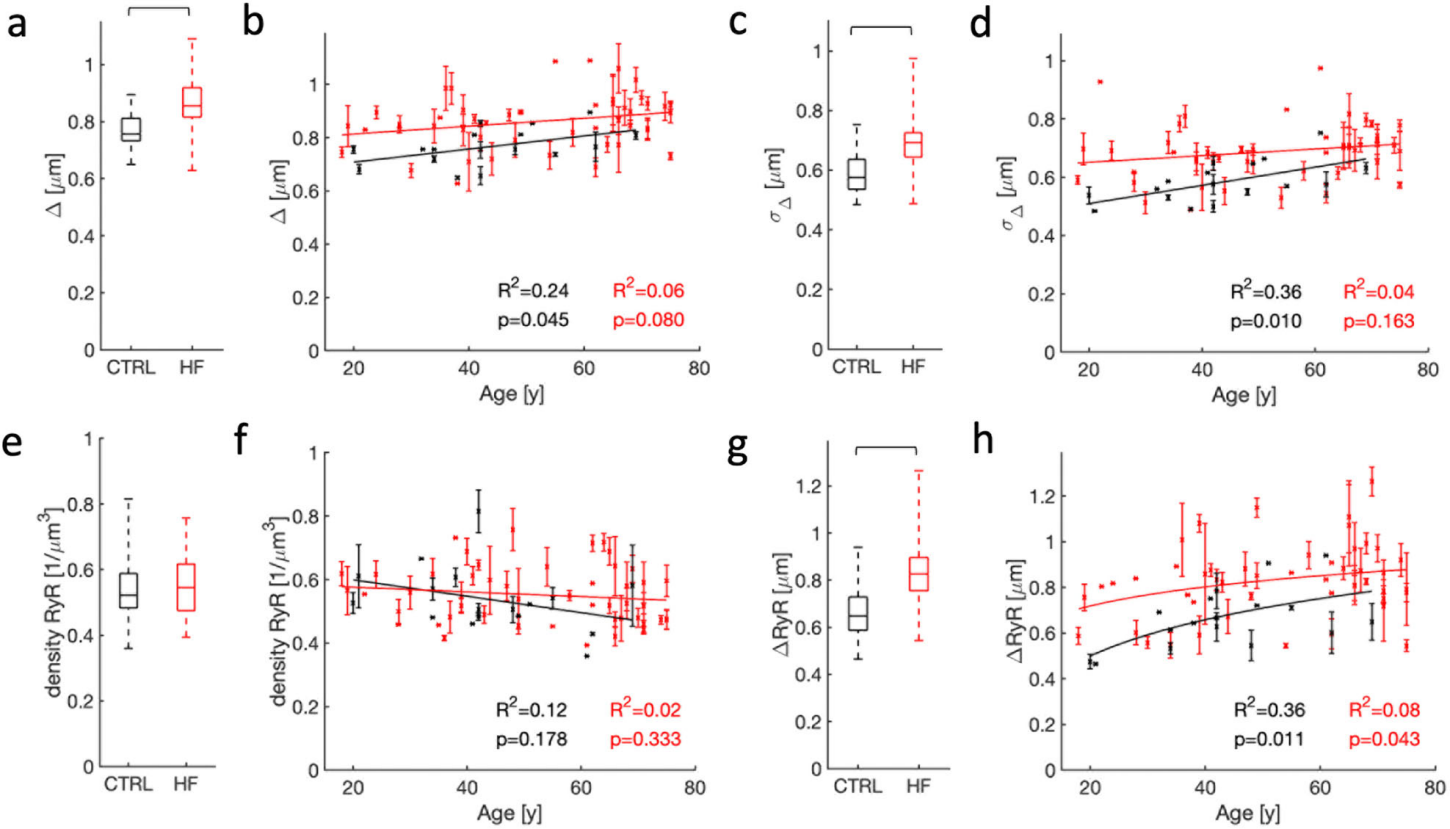
Statistical analyses of age- and HF-associated remodeling of the t-system and RyR clusters. **a** Sarcolemmal distances were smaller in cardiomyocytes from donors than HF patients. **b** Effects of aging on the sarcolemmal distance were small in donors and not significant in HF patients. **c** The standard deviation of sarcolemmal distances was smaller in cardiomyocytes from donors than HF patients. **d** Standard deviations of the sarcolemmal distances were affected by age in donors. Effects were not significant in HF patients. **e** The density of RyR clusters was not different in donors and HF patients. **f** RyR cluster density was not affected by age. **g** The distance of RyR clusters to the sarcolemma was smaller in donors than HF patients. **h** Aging was associated with an increasing sarcolemmal distance of RyR clusters in donor cells. Effects of age on this distance were small in HF patients.

Based on analyses of the image stacks in the library, we explored the relationship between age and microstructural features using linear and nonlinear regression models in donors and HF patients (Fig. 5). From 3D reconstructions of the sarcolemma including t-system, we extracted the sarcolemmal distances of intracellular regions. The range of these distances and the average distance were increased in HF vs. control (Fig. 5a). Effects of age on the sarcolemmal distance were present but small in donors (Fig. 5b), whereas age did not affect the sarcolemmal distances in HF patients. As a further measure of the heterogeneity of the t-system, we investigated the standard deviation of sarcolemmal distances of intracellular regions. Similar to the average sarcolemmal distances, their standard deviation exhibited an increased range and increased average in HF vs. control (Fig. 5c). Age had a medium effect on the standard deviation of the sarcolemmal distance in donors but did not affect this measure in HF patients (Fig. 5d).

We subsequently investigated if age affects RyR cluster organization. Differences in the density of RyR clusters in donors and HF patients were not significant (Fig. 5e), neither did age affect the density (Fig. 5f). However, the range and average of sarcolemmal distances of RyR clusters were increased in HF vs. control (Fig. 5g), which can be considered as a direct consequence of the reduction in t-system density observed in HF. We also found that RyR-sarcolemma distance increased with age in myocytes from both donors and HF patients (Fig. 5h). A summary of the statistical analyses is presented in Supplementary Tables 1 and 2. The analyses show that remodeling of structures crucial for efficient EC coupling in failing hearts is more heterogeneous and often more severe than in the aging heart. Nevertheless, remodeling of these structures appears to be also a hallmark of aging.

### Loss of density and sarcolemmal association of JPH2 clusters with aging

**Fig. 6:**
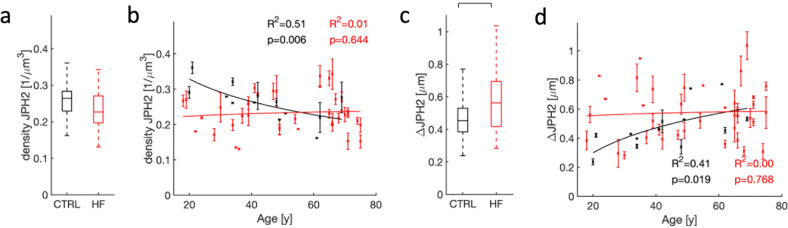
Statistical analyses of remodeling of JPH2 clusters and their association with the sarcolemma. **a** Differences in JPH2 cluster density between donor and HF patients were not significant. **b** Aging was associated with a decreasing density of JPH2 clusters in donors. Effects of age on cluster density were not significant in HF patients. **c** JPH2 clusters were more proximal to the sarcolemma in donors versus HF patients. **d** Aging was associated with an increased sarcolemmal distance of JPH2 clusters in donors. Age effects on the sarcolemmal distance were not significant in HF patients.

Studies on rodent models suggest an important role of JPH2 in postnatal formation and maintenance of t-system and its relationship to the SR^16,41,42^. Therefore, we used the image library to investigate if JPH2 remodeling can explain age and HF-associated remodeling of the t-system. We did not find differences in the density of JPH2 clusters between control and HF myocytes (Fig. 6a). However, the cluster density strongly decreased with age in donor cells (Fig. 6b). Based on a comparison of p-values, a logarithmic model described the decrease better than the linear model. The range and average of sarcolemmal distances of JPH2 cluster were increased in HF vs. control (Fig. 6c). The distances increased with age in donor cells (Fig. 6d), approaching average values similar to HF at ages over 60 years. A logarithmic model was superior vs. a linear model in describing the increases. Age did not affect the density and sarcolemmal distance of JPH2 clusters in cells from HF patients.

### Effects of JPH2 density on RyR localization

Because JPH2 was suggested to be crucial for couplon formation by tethering the SR and sarcolemma^15,16^, we investigated if the reduction in JPH2 density in donor hearts with age (Fig. 6b) affects localization of RyR clusters. We introduced measures of fractional sarcolemmal association of RyR and JPH2 clusters (F_RyR_/F_SL_ and F_JPH2_/F_SL_, respectively) by calculating the ratio of cluster volume and intracellular volume within 250 nm of the sarcolemma. With these measures, a value of 0% indicates that clusters are not sarcolemma associated. A value of 100% suggests that the spatial density of clusters is similar within the cell and sarcolemma, and the absence of a preferential localization. However, values >100% indicate a preference for the sarcolemmal association of clusters. In particular, high F_RyR_/F_SL_ is expected for couplons.

**Fig. 7:**
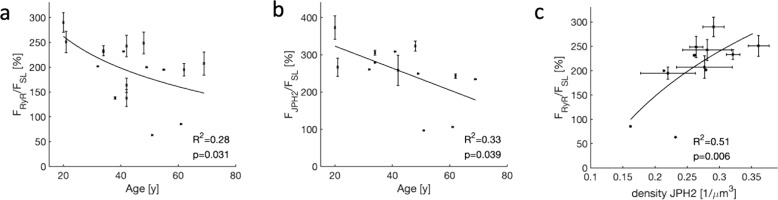
Statistical analyses of the fractional sarcolemmal association of RyR and JPH2 clusters in donor cardiomyocytes. Aging was associated with a decreasing fractional sarcolemmal association of **a** RyR and **b** JPH2 clusters. **c** The density of JPH2 clusters strongly affected the fractional sarcolemmal association of RyR clusters. Increased JPH2 cluster density was associated with a higher fractional association of RyR clusters.

Our analyses revealed a decreasing relationship of F_RyR_/F_SL_ and F_JPH2_/F_SL_ with age (Fig. 7a, b). This indicates that in cells from young donors, RyR and JPH2 clusters were located preferentially at the sarcolemma. In contrast, in cells from old donors, such a preference was hardly visible. The density of JPH2 clusters was highly associated with F_RyR_/F_SL_ (Fig. 7c), which suggests that reduction of JPH2 density (Fig. 6b) underlies decreased sarcolemmal localization of RyR clusters and thus couplon formation with age. Similar analyses in HF hearts did not indicate relationships as described for donors (Supplementary Table 2).

### Occurrence of large autofluorescent regions in aging and HF cardiomyocytes

To relate microstructural remodeling to the presence of lipofuscin, we quantified the volume fraction of large autofluorescent regions (*V*_633,large_/*V*_633_) exemplified in Fig. 3c and Supplementary Fig. 1. *V*_633,large_/*V*_633_ of 0% marked the absence of autofluorescent regions. A value of 100% reflects that all signal is within regions with a volume ≥1 µm^3^. These regions occurred in both donor and HF cells (Supplementary Fig. 1). We found an increase in *V*_633,large_/*V*_633_ with age in donor cells (Supplementary Fig. 2b). The increase was best described with a linear model and is in agreement with other studies describing the accumulation of lipofuscin with age in cardiomyocytes^37^. The effect of age on *V*_633,large_/*V*_633_ was not significant in HF cells. *V*_633,large_/*V*_633_ was not associated with remodeling of t-system (Supplementary Fig. 2c, d) or RyR clusters (Supplementary Fig. 2e, f), but showed a linear and negative relationship with the density of JPH2 clusters (Supplementary Fig. 2g). This applied not only to donors, but also to HF cells. Similarly, increases in *V*_633,large_/*V*_633_ were associated with increased sarcolemmal distances of JPH2 clusters in both donor and HF cells (Supplementary Fig. 2h). The regression analyses are summarized in Supplementary Tables 2 and 3.

### Effects of age on gene and protein expression

We investigated if age affects gene expression of RyR and JPH2, using quantitative polymerase chain reaction (qPCR) on the left ventricular myocardium from a subset of the donors (Supplementary Fig. 3). The subset covered nine donors with an age ranging from 21 to 62 years. Regression analysis of measured differences in threshold cycle between the target genes and a reference gene, ΔCT, revealed negligible effects of age on the expression of RyR and JPH2. Usage of linear or logarithmic models did not improve fitting vs. usage of constant models.

Similarly, we used western and dot blot analysis to assess protein expression in nine donors with an age of 20–69 years. Age did not affect RyR expression (Supplementary Fig. 4). However, the effect on JPH2 expression was strong (Supplementary Fig. 5). A logarithmic model was superior vs. a linear model in describing the decrease of JPH2 expression. This supported the finding of an age-related decrease in JPH2 cluster density shown in Fig. 6.

### Multi-faceted age-associated remodeling at the nanometer scale

We provide examples for the multi-faceted age-associated remodeling at the nanometer scale in Supplementary Fig. 6. We applied transmission electron microscopy (TEM) on LV tissue samples from a young (31 years) and an old donor (62 years). Supporting our findings in Fig. 5, the t-system in tissue from the young donor appeared denser and more organized vs. the old donor. The TEM images support prior work in animal models of aging of cardiac and skeletal myocytes that revealed, for instance, age-associated remodeling of mitochondria^43,44^. Furthermore, we found regions of high density, arguably, lipofuscin, in the images from old donors (Supplementary Fig. 7).

## Discussion

Our study provides new insights into the remodeling of structures and proteins linked to EC coupling in cardiomyocytes, in the aging human heart. We introduced a library of 3D reconstructions of the microstructure of left ventricular myocardium from donors and HF patients and applied the library to elucidate multi-faceted remodeling during aging. Our study revealed that the t-system and its association with RyR clusters diminish with age towards a phenotype found in HF cardiomyocytes. Importantly, the extent of this age-associated remodeling remains less than in advanced chronic HF. Nevertheless, based on a previously developed model of EC coupling^45^, effects of the displayed age-associated remodeling are loss of sites for calcium-induced calcium release in regions distal to the sarcolemma including t-system. This loss causes increased spatiotemporal heterogeneity of the calcium transient. Beyond this model, studies on tissues from human failing hearts suggest that the degree of t-system remodeling predicts the negative-force frequency relationship and time to relaxation^27^.

In an attempt to elucidate the mechanisms of remodeling, we provided evidence that JPH2 clusters, which tie the SR and sarcolemma to form couplons, exhibited a smaller density and larger sarcolemmal distance with increasing age. An effect of HF on the density of JPH2 clusters was not observed, but sarcolemmal dissociation of the clusters was similar for HF and old age. It is currently unclear if these dissociated JPH2 clusters might traffic to form junctional complexes and couplons or have other functional roles. A limitation of our study is that we did not assess LCCs and thus cannot make comprehensive statements on couplon formation.

Further inspection of the image stacks allowed us to assess the accumulation of autofluorescent lipofuscin in perinuclear regions in the aging heart. In agreement with prior work^37^, age strongly increased our measure of lipofuscin. Lipofuscin was also present in some HF hearts, but not a consistent feature of HF. We speculate that the presence of lipofuscin can serve as a marker for microstructural remodeling in the aging, non-failing heart, in particular, of JPH2.

Our studies suggest that JPH2 gene expression does not explain the reduction of JPH2 protein level and cluster density with age. Alternatively, deficiencies in trafficking and related processes, such as protein recycling or autophagy, could cause these reductions. Deficiencies of trafficking of JPH2 clusters, accompanied by t-system remodeling and calcium handling dysfunction, were identified previously in a mouse model of pressure-overload induced cardiomyopathy and human cardiomyopathy^46^. The trafficking deficiencies were explained by the densification of microtubules. Their depolymerization attenuated t-tubule remodeling. In isolated mouse cardiomyocytes and myocardial sections from patients with ischemic and dilated cardiomyopathy, the JPH2 distribution was remodeled into irregular punctations and aggregations. In cultured mouse and also in rat cardiomyocytes^47^, JPH2 was located away from the t-system in the cell periphery. We did not find this redistribution in cells from old donors, but a major aspect of JPH2 remodeling, i.e., the reduced density of JPH2 clusters, is similar. An alternative explanation for reduced JPH2 protein level and cluster density is increased JPH2 degradation. In the diseased heart, decreased expression of JPH2 has been previously explained by cleavage of JPH2 by the calcium-dependent protease calpain^48^. Cleaved JPH2 was found to be non-functional for normal calcium-induced calcium release. A role of JPH2, beyond formation and maintenance of the t-system and couplons, as a stress-adaptive transcription regulator in cardiomyocytes was suggested. The N-terminal fragment of JPH2 after stress-induced proteolysis was found to translocate to the nucleus and alter the transcriptional profile. Overexpression and loss of the fragment attenuated and accelerated pathological remodeling, respectively, in response to cardiac stress^49^. A different role for a cleaved C-terminal fragment was proposed^50^. The fragment was elevated in the mouse models of HF and HF patients. Stress-induced remodeling was reduced, when the nuclear localization sequence in the fragment was mutated. Our studies did not provide evidence for nuclear localization of JPH2 in donor and HF hearts but indicate that JPH2 remodeling plays nevertheless an important role in aging cardiomyocytes. Extending concepts developed in studies of the role of JPH2 in heart disease^18,51–53^, we suggest that targeting JPH2 expression and degradation can serve as a remedy against age-associated t-system and RyR remodeling. We also propose that our study establishes a basis for investigations of functional changes in aging human ventricular myocytes and age-associated aggravation of heart diseases.

## Methods

### Study population and tissue acquisition

All studies were performed in accordance with relevant guidelines and regulations. The studies and methods were approved by the institutional review boards of the University of Utah Health, Intermountain Medical Center, Salt Lake City VA Medical Center, which are members of Utah Transplantation Affiliated Hospitals (U.T.A.H.) Cardiac Transplant Program. Enrollment in this study has been described earlier^26,54^. Samples from the left ventricular free wall were obtained from 17 organ donors without a history of cardiac disease. Furthermore and after their written informed consent, 49 patients with advanced HF (New York Heart Association classes III or IV) were enrolled and provided tissue samples at the time of LVAD implantation (*n* = 42) or heart transplantation without prior LVAD implantation (DTX, *n* = 7). For all patients and donors, LVEF was measured using standard echocardiography. Furthermore, clinical data were collected.

### Tissue sectioning and labeling

After excision from the left ventricle, tissue samples were snap-frozen in optical cutting temperature (OCT) compound (Sakura Finetek Europe BV, Alphen aan den Rijn, Netherlands) and stored at −80 °C. The samples were sectioned at 100 µm thickness with a cryotome (CM1950, Leica, Wetzlar, Germany), immediately immersed in 1% PBS for 10–15 min. The sections were subsequently fixed in 1% paraformaldehyde for 10 min and then washed in PBS. Also, left ventricle tissue samples were collected and fixed in 2% PFA (FB002, Thermo Fisher Scientific, IL) for 4–24 h. Fixed tissue samples were sectioned into slices of 100 µm thickness using a vibratome (Leica VT1200S, Germany). Slices were washed three times with 1X PBS (Thermo Fisher Scientific) for 10 min after every labeling step on a shaker. Slices were incubated with 0.5% Triton-X-100 (T8787, Sigma Aldrich, MO) for 2 h, followed by signal enhancer for 30 min and blocking buffer made up of 0.05% Triton-X 100 and 10% donkey serum (D9663, Sigma Aldrich, MO) for 90 min. Slices were immunolabeled with primary RyR2 monoclonal (MA3-916, Fisher Scientific) and JPH2 polyclonal (40-5300, Fisher Scientific) antibodies in the ratio of 1:100 overnight on a shaker. Next day sections were labeled in dark with secondary antibodies conjugated to Alexa Fluor 633 goat anti-rabbit IgG (A21070, Invitrogen, MA) and Alexa Fluor 488 goat anti-mouse IgG (A11001, Invitrogen) in the ratio of 1:100 for 3 h. Incubated with nuclear stain DAPI at 1 μg/ml for 15 min and WGA conjugated to Alexa Fluor 555 (W32464, Thermo Fisher Scientific) at 30 μg/ml for 4 h. Tissue slices were mounted on a glass slide, embedded in Fluoromount-G (17984-25, Electron Microscopy Science, PA), and then dried for at least 24 h at room temperature and at <40% relative humidity.

### Confocal microscopy

Labeled sections were imaged using a confocal microscope (TCS SP8, Leica, Jena, Germany) equipped with a 60× oil immersion lens. Images were acquired from regions with a high myocyte content. Three-dimensional image stacks were acquired within 25 µm from the coverslip. The dimension of the stacks was 1024 pixels in *x* and *y* direction and ranged between 100 and 300 slices in the *z*-direction. The length, width, and height of voxels was 0.1 µm. The imaging protocol was configured to collect signals from DAPI, RyR, WGA, and JPH2 associated fluorescence. The fluorophores were excited by lasers of wavelengths 405, 488, 561, and 633 nm, respectively.

### Image processing

We processed and analyzed the 3D image stacks using Matlab (R2019a and later, Mathworks, Natick, MA) similarly as described in our prior work for various species^55,56^. All image processing and the figure generation for this study were implemented in Matlab scripts to assure reproducibility. In short, images exhibiting weak signal-to-noise ratios or microstructural deterioration were excluded from the analyses. We deconvolved the image stacks using the Richardson-Lucy algorithm and corrected for depth-dependent attenuation. Regions outside of tissue were identified based on thresholding and excluded for the subsequent processing. The image stacks of WGA and DAPI labeling were segmented in nuclear, intracellular, and extracellular regions by histogram-based thresholding and morphological operators. As measures of t-system density and regularity, we quantified the mean and standard deviation of the distance between the sarcolemma and intracellular sites. We applied distance maps to efficiently calculate sarcolemmal distances.

RyR and JPH2 clusters were extracted by two-pass histogram-based thresholding to account for the presence of perinuclear large autofluorescent regions. A first pass histogram-based thresholding allowed us to segment the clusters and autofluorescent regions. We defined a size of 1 µm^3^ to reliably identify the autofluorescent regions, which were then excluded in the second pass histogram-based thresholding for segmentation of the clusters only.

The volume fraction of large autofluorescent regions was determined from the volume of the autofluorescent regions (*V*_633,large_) divided by the volume of thresholded regions (*V*_633_) extracted in the first pass from the JPH2 images.

We calculated RyR and JPH2 cluster densities as the number of clusters per unit volume. The relationship of clusters to the t-system was statistically characterized by evaluating the histograms of the sarcolemmal distance of clusters.

As a measure of the preference for the sarcolemmal association of RyR and JPH2 clusters, we assessed the region within 250 nm of the sarcolemmal versus the complete intracellular volume. We determined the ratio of fractional cluster volumes (F_RyR_ and F_JPH2_, respectively) and the fractional proximal intracellular volume (F_SL_).

### RNA extraction and quantitative PCR analysis

Total RNA was extracted from the left ventricular myocardium of donor hearts in Tri reagent (T9424, Sigma Aldrich Inc, GA) and using miRNeasy Mini kit (217004, Qiagen, Germantown, MD) according to the manufacturer’s protocol. RNA integrity was confirmed using NanoDrop 2000 spectrophotometer (Thermo Fisher Scientific, Waltham, MA). cDNA was synthesized using the QuantiNova reverse transcription cDNA synthesis kit (205411, Qiagen) following the manufacturer’s protocol. TaqMan® gene expression assays used in the study were GAPDH (Hs02786624_g1, Thermo Fisher Scientific) and GUSB (Hs00939627_m1, Thermo Fisher Scientific) as primary and secondary housekeeping genes, while RyR2 (Hs00181461_m1, Thermo Fisher Scientific) and JPH2 (Hs00375310_m1, Thermo Fisher Scientific) constituted the target genes. Gene expression was analyzed by qPCR using TaqMan Fast Advanced Master Mix (4444556, Thermo Fisher Scientific) and TaqMan gene expression assays (Applied Biosystems, Foster City, CA) on Applied Biosystems™ QuantStudio™ 12K Flex Real-Time PCR Detection machine. We determined the difference (ΔCT) between cycle thresholds for GAPDH and the other genes. ΔCT is the difference in threshold cycle between the target and reference genes: ΔCT = CT (a target gene) − CT (housekeeping gene). Regression analyses were performed to assess relationships between age and ΔCT of RyR2 and JPH2.

### Protein extraction and western blotting

LV tissue samples were collected from donor hearts ranging from 20 to 75 years of age. The tissue sample was snap-frozen in liquid nitrogen and stored at −80 °C. In all, 30 mg of tissue sample was homogenized using metal beads for 5 min in RIPA buffer with 2x protease and phosphatase inhibitor (Thermo Scientific, 78440) at 4 °C. The homogenate was transferred to a new tube containing 10 µl of 1x PMSF and was allowed to rotate for 30 min at 4 °C and then centrifuged for 10 min at 4 °C. The supernatant was used for protein estimation using the Pierce BCA Protein Assay kit (Thermo Scientific, 23225). An equal volume of 2x Laemmeli buffer with 10% DTT was added to the sample and boiled for 10 min at 98 °C. In all, 30 mg protein was used for SDS-PAGE. The gels ran at constant volts (250 V) and then the proteins were transferred to a nitrocellulose membrane at constant current (350 mA). The membranes were blocked for 1 h in 5% non-fat milk and probed with primary antibodies overnight in the cold room. The primary antibodies used at 1:1000 dilution were JPH2 (405300, Thermo Fisher) and GAPDH (5174, Cell Signaling Technology, Danvers, MA). Blots were then washed with 1x TBST three times for 10 min and probed with secondary antibody at 1:10,000 dilution for 1 h. Blots were washed with 1x TBST three times before scanning using LI-COR. Image Studio Lite (LI-COR Biosciences, Lincoln, NE) was used to analyze the western blots. The presented blots derive from the same experiment and were processed in parallel.

### Dot blotting

The same protein lysates were used as above for the western blot experiment. 2 µl of protein lysates were pipetted on a nitrocellulose membrane for each sample. The membrane was then blocked in 5% BSA for 1 h followed by washing using PBST three times for 10 min each. Blot was incubated in primary antibody at 1:1000 dilution for RyR (MA3-916, Invitrogen) and GAPDH (ab37168, Abcam) for 1 h. The membrane was washed by PBST three times for 10 min each and labeled with IRDye^®^ 680RD donkey anti-mouse IgG secondary antibody and IRDye^®^ 800CW donkey anti-mouse IgG secondary antibody (LI-COR Biosciences) both at 1:10000 dilution for 1 h. Blots were washed before imaging. Image Studio Lite was used to analyze the dot blots. The presented blots derive from the same experiment and were processed in parallel.

### TEM of cardiac tissues

LV tissue samples were collected from donors hearts and fixed in fixation solution (2.5% glutaraldehyde, 1% paraformaldehyde, and 0.1 M sodium cacodylate buffer) overnight at 4 °C. The next day, washed with the cacodylate buffer two times for 10 min. The tissue was post-fixed in 2% osmium tetroxide buffer for 2 h and rinsed in nanopore water for 5 min followed by en-bloc staining for 1 h at room temperature while rotating with saturated aqueous uranyl acetate filtered with 0.22 µm pore size Millipore syringe filter. The tissues were then dehydrated through a graded series of ethanol: 50% once for 10 min, 70% once for 10 min, 95% twice for 10 min each, 100% ethanol four times for 10 min each, and with absolute acetone three times for 10 min each. Infiltration was performed by incubating the samples at room temperature and gradually increasing the concentration of epoxy resin (Electron Microscopy Sciences, 14900, Hatfield, PA). Samples were transferred to 50% resin in acetone for 1 h and 75% resin in acetone overnight. Samples were then transferred to 100% resin for 8 h, three changes with fresh resin, and embedded and polymerized for 48 h at 60 °C. In all, 70-nm-thick sections were obtained using the Leica UC 6 (Leica Microsystems, Vienna, Austria) and collected on 200-mesh copper grids. Sections were counterstained with saturated uranyl acetate for 10 min followed by staining with lead citrate for 5 min. Sections were viewed on a JEM 1400 Plus transmission electron microscope (JEOL, Peabody, MA) at 120 kV. Images were acquired on a Gatan 2Kx2K digital camera.

### Statistical analyses

Analyses were performed using Matlab. Repeated measurements for donors and patients were averaged for statistical analyses. Statistical data were expressed as means±standard error or means ± standard deviation. Multigroup comparisons were performed using one-way analysis of variance (ANOVA) and the Tukey-Kramer post-hoc test. Two-group comparisons were performed with unpaired t-tests. In general, we considered p < 0.05 as significant.

For regression analyses, we defined linear and logarithmic models:

1 $${\mathrm{y}}({\mathrm{x}}) = {\mathrm{a}} + {\mathrm{bx}}$$

2 $${\mathrm{y}}({\mathrm{x}}) = {\mathrm{a}} + {\mathrm{b}}\,\log ({\mathrm{x}})$$

with the free parameters a and b. We assessed the models with the Matlab functions fitlm and fitnlm. Differences of the models versus constant models were considered significant if *P* < 0.05. Presentation of regression analyses was limited to the model yielding the minimal *P*. The coefficient of determination R^2^ determined the goodness of model fit to experimental data. In boxplots, the central mark, bottom, and top box edges identified the median, 25th, and 75th percentile, respectively. Whiskers described the data range.

### Reporting summary

Further information on research design is available in the Nature Research Reporting Summary linked to this article.

## Supplementary information

Supplemental Materail

Movie 1

Movie 2

Movie 3

Reporting Summary

## Data Availability

The data that support the findings of this study are available from the corresponding author upon reasonable request.

## Code availability

The code for processing and analyses in this study is available from the corresponding author upon reasonable request.
