# Supplementary material for: Remodeling of t-system and proteins underlying excitation-contraction coupling in aging versus failing human heart
Source: NPJ Aging Mech Dis. 2021 May 28;7:16. doi: 10.1038/s41514-021-00066-7 (PMC8163749; doi:10.1038/s41514-021-00066-7)
Supplement: Supplementary file 1 — Supplemental Materail [file 41514_2021_66_MOESM1_ESM.pdf]

Supplementary Files

Remodeling of the Transverse Tubular System and Proteins Underlying Excitation-Contraction Coupling in the Aging Versus Failing Human Heart

Yankun Lyu, MD, PhD, Vipin K. Verma, PhD, Younjee Lee, Iosif Taleb, MD, Rachit Badolia, Thirupura S. Shankar, Christos P. Kyriakopoulos, MD, Craig H. Selzman, MD, William Caine, MD, Rami Alharethi, MD, Sutip Navankasattusas, PhD, Thomas Seidel, MD, PhD, Stavros G. Drakos, MD, PhD, Frank B. Sachse, PhD

Supplementary Tables

Supplementary Table 1. Statistics of measures from donor and HF patients.

Measure	Unit	Donor (mean±stddev)	HF (mean±stddev)	p
Sarcolemmal distance	μm	0.766±0.068	0.861±0.100	0.001
Stddev of sarcolemmal distance	μm	0.584±0.070	0.688±0.096	0.000
Density of RyR clusters	$1/\mu m^3$	0.538±0.102	0.552±0.090	0.600
Sarcolemmal distance of RyR clusters	μm	0.666±0.132	0.827±0.161	0.000
Density of JPH2 clusters	$1/\mu m^3$	0.264±0.050	0.233±0.055	0.085
Sarcolemmal distance of JPH2 clusters	μm	0.475±0.150	0.575±0.188	0.088
Volume fraction of regions with autofluorescence for 633 nm illumination	%	42.405±9.234	31.835±17.538	0.074

Supplementary Table 2. Regression analyses of relationships of age and microstructural measures from donor and HF patients.

Variable x	Variable y	Group	Model	a	b	R^2	p
Age	Sarcolemmal distance	Donor	(a1 + a2*x)	0.659	0.002	0.241	0.045
		HF	(a1 + a2*x)	0.784	0.001	0.064	0.080
	Stddev of sarcolemmal distance	Donor	(a1 + a2*x)	0.447	0.003	0.365	0.010
		HF	(a1 + a2*x)	0.629	0.001	0.041	0.163
	Sarcolemmal distance of RyR clusters	Donor	(a1 + a2*log(x))	-0.186	0.229	0.356	0.011
		HF	(a1 + a2*log(x))	0.353	0.121	0.084	0.043
	Density of RyR clusters	Donor	(a1 + a2*x)	0.651	-0.003	0.118	0.178
		HF	(a1 + a2*x)	0.591	-0.001	0.020	0.333
	Sarcolemmal distance of JPH2 clusters	Donor	(a1 + a2*log(x))	-0.439	0.247	0.405	0.019
		HF	(a1 + a2*log(x))	0.487	0.023	0.003	0.768
	Density of JPH2 clusters	Donor	(a1 + a2*log(x))	0.605	-0.092	0.510	0.006
		HF	(a1 + a2*log(x))	0.192	0.011	0.006	0.644
	$V_{633,large}/V_{633}$	Donor	(a1 + a2*x)	-0.512	0.989	0.614	0.002
		HF	(a1 + a2*x)	22.770	0.176	0.032	0.288
	LVEF [%]	Donor	(a1 + a2*x)	53.827	0.191	0.100	0.217
		HF	(a1 + a2*x)	15.920	0.076	0.029	0.296
	F_{RyR}/F_{SL} [%]	Donor	(a1 + a2*log(x))	538.834	-92.313	0.275	0.031
		HF	(a1 + a2*log(x))	207.187	-4.979	0.002	0.782
	F_{JPH2}/F_{SL} [%]	Donor	(a1 + a2*x)	382.444	-2.953	0.334	0.039
		HF	(a1 + a2*x)	278.450	-0.266	0.004	0.701

Supplementary Table 3. Regression analyses of relationships of $V_{633,large}/V_{633}$ and microstructural measures from donor and HF patients.

Variable x	Variable y	Group	Model	a	b	R^2	p
$V_{633,large}/V_{633}$	Sarcolemmal distance	Donor	(a1 + a2*x)	0.734	0.001	0.163	0.171
		HF	(a1 + a2*x)	0.868	0.000	0.004	0.695
	Stddev of sarcolemmal distance	Donor	(a1 + a2*x)	0.520	0.002	0.254	0.079
		HF	(a1 + a2*x)	0.678	0.001	0.026	0.336
	Sarcolemmal distance of RyR clusters	Donor	(a1 + a2*log(x))	0.343	0.087	0.190	0.136
		HF	(a1 + a2*log(x))	0.871	-0.011	0.003	0.760
	Density of RyR clusters	Donor	(a1 + a2*x)	0.576	-0.001	0.116	0.255
		HF	(a1 + a2*x)	0.569	-0.001	0.012	0.516
	Sarcolemmal distance of JPH2 clusters	Donor	(a1 + a2*log(x))	0.041	0.121	0.354	0.032
		HF	(a1 + a2*x)	0.462	0.004	0.111	0.044
	Density of JPH2 clusters	Donor	(a1 + a2*x)	0.332	-0.002	0.381	0.025
		HF	(a1 + a2*x)	0.283	-0.002	0.246	0.002

Movie Legends

Movie 1. 3D reconstruction of segment of ventricular cardiomyocyte from 21-year old donor.

Movie 2. 3D reconstruction of segment of ventricular cardiomyocyte from 69-year old donor.

Movie 3. 3D reconstruction of segment of ventricular cardiomyocyte from 19-year old patient with chronic HF undergoing LVAD implantation.

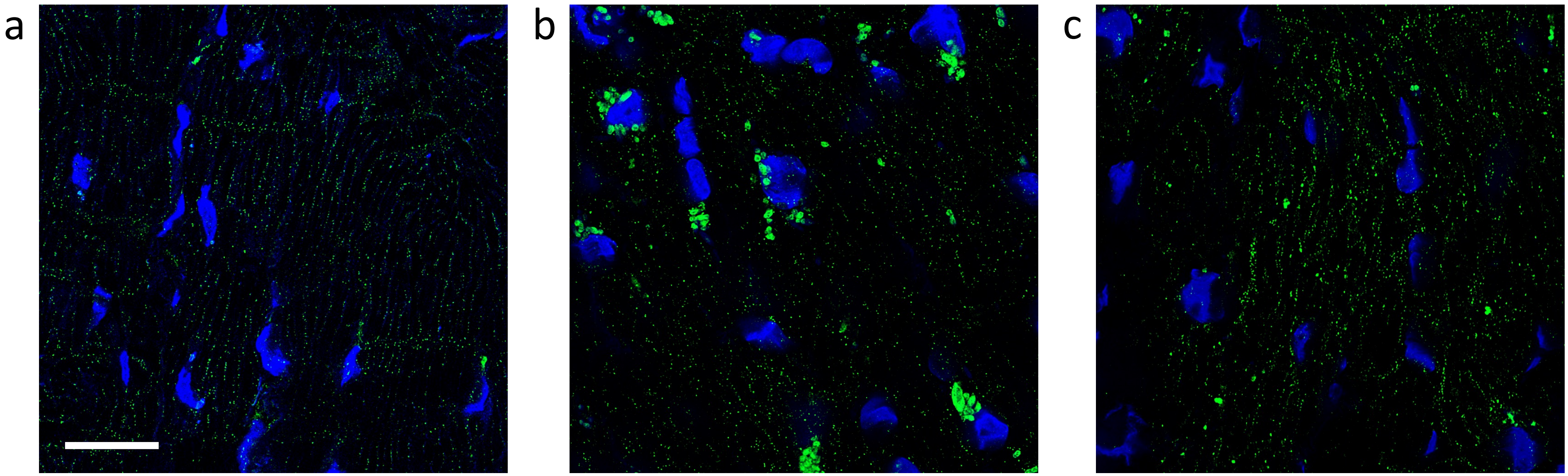

Supplementary Figure 1. Confocal microscopy of left ventricular myocardium from (a) 21-year and (b) 69-year old donor as well as (c) 19-year old LVAD patient. Tissue was labelled with DAPI (blue) and anti-JPH2 antibodies (green). In tissue from the 69-year old donor, large autofluorescent regions were located in the perinuclear region. Autofluorescent regions of this degree were not present in tissue from the young donor and HF patient. Scale bar in (a) marks 20 μm and applies to (b) and (c).

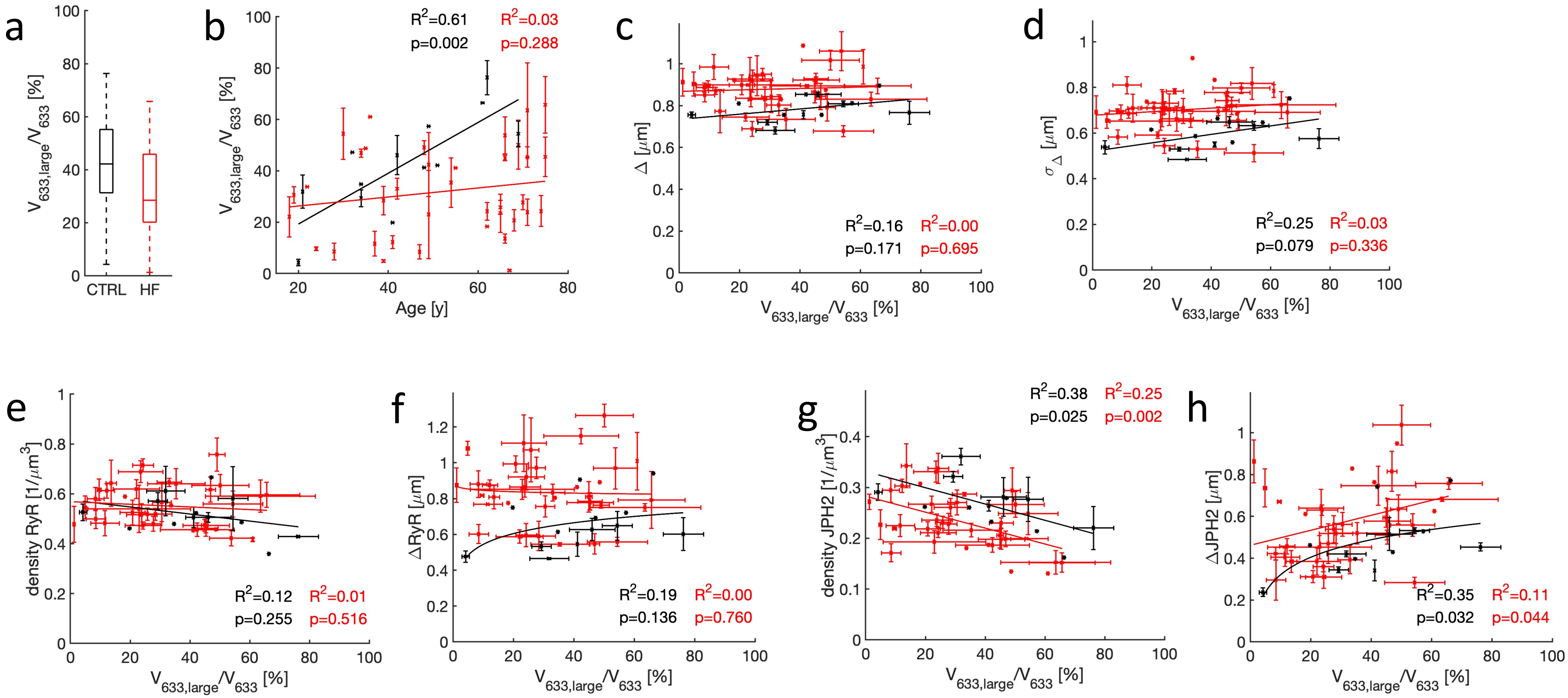

Supplementary Figure 2. Analysis of the volume fraction of large regions in response to 633 nm illumination ($V_{633,large}/V_{633}$) and microstructural features. (a) $V_{633,large}/V_{633}$ was higher in donors than HF patients. (b) With increased age, $V_{633,large}/V_{633}$ increased in donor cells. In HF cells, effects of age on $V_{633,large}/V_{633}$ were not significant. Effects of $V_{633,large}/V_{633}$ on (c) sarcolemmal distance and (d) stddev of sarcolemmal distance as well as (e) density and (f) sarcolemmal distance of RyR clusters were not significant. Increased $V_{633,large}/V_{633}$ was associated with (g) decreased density and (h) increased sarcolemmal distance of JPH2 clusters.

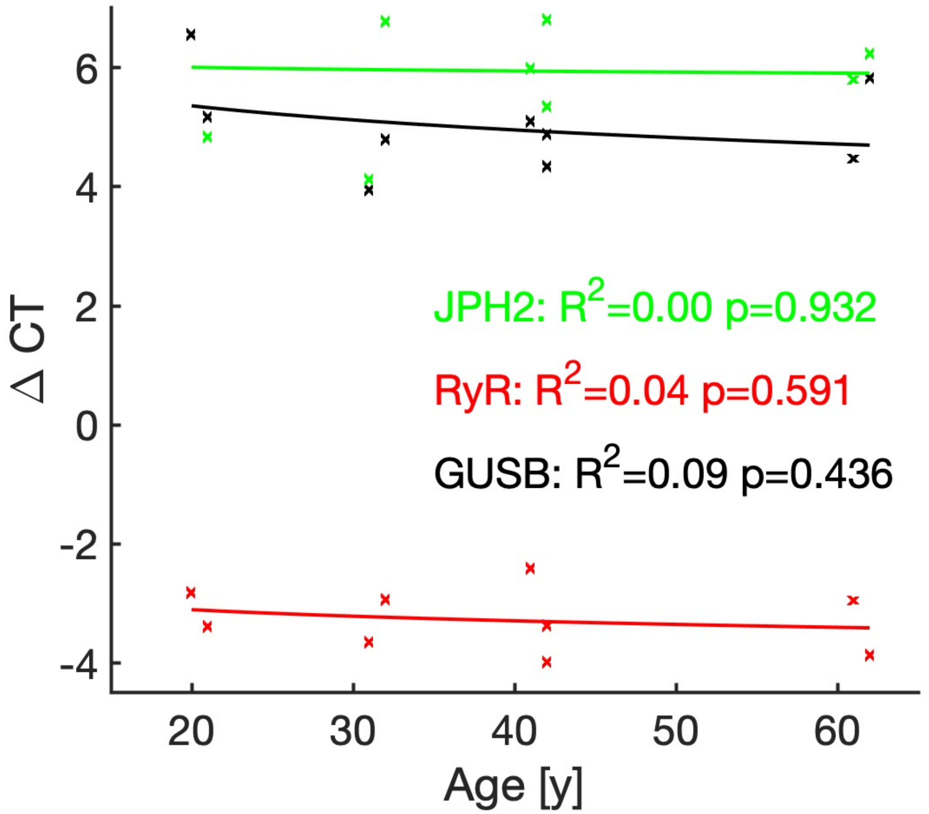

Supplementary Figure 3. Effects of aging on gene expression in ventricular myocardium measured with qPCR. GAPDH was used as the housekeeping gene. GUSB served as a secondary housekeeping gene. The effect of age with RyR and JPH2 expression was negligible. Differences of linear and logarithmic models versus a constant model of the relationship were not significant.

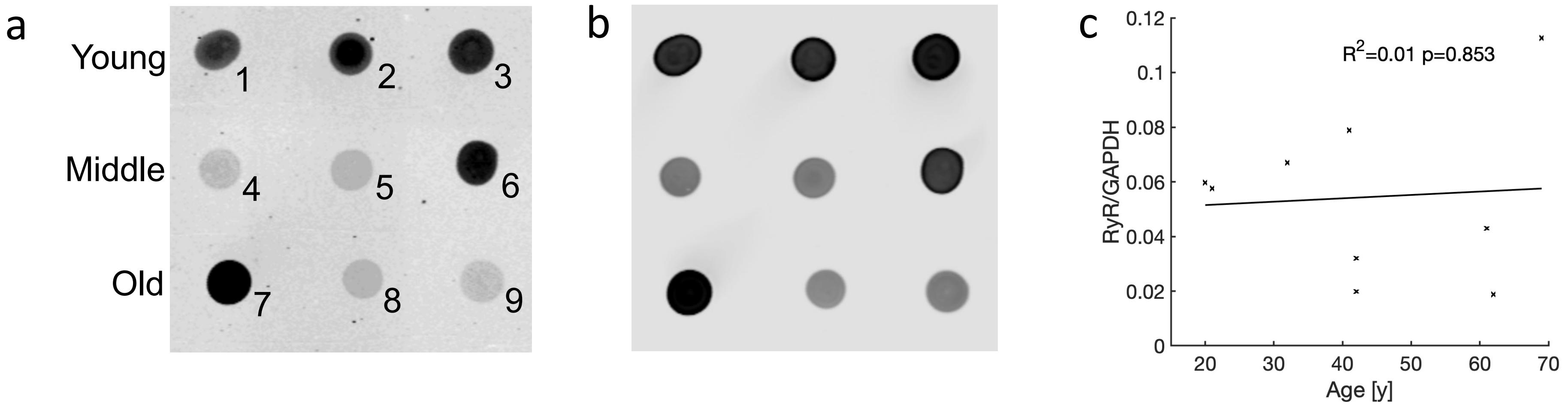

Supplementary Figure 4: RyR expression in aging donor hearts. Dot blot for expression of (a) RyR and (b) GAPDH. First row, sample 1-3 were from young donors (age<35). Second row, sample 4-6 were from middle age donors (aged 35-55). Third row, samples 7-9 were from old donors (age>55). (c) Quantification of RyR expression. Differences of RyR expression for different ages were not significant.

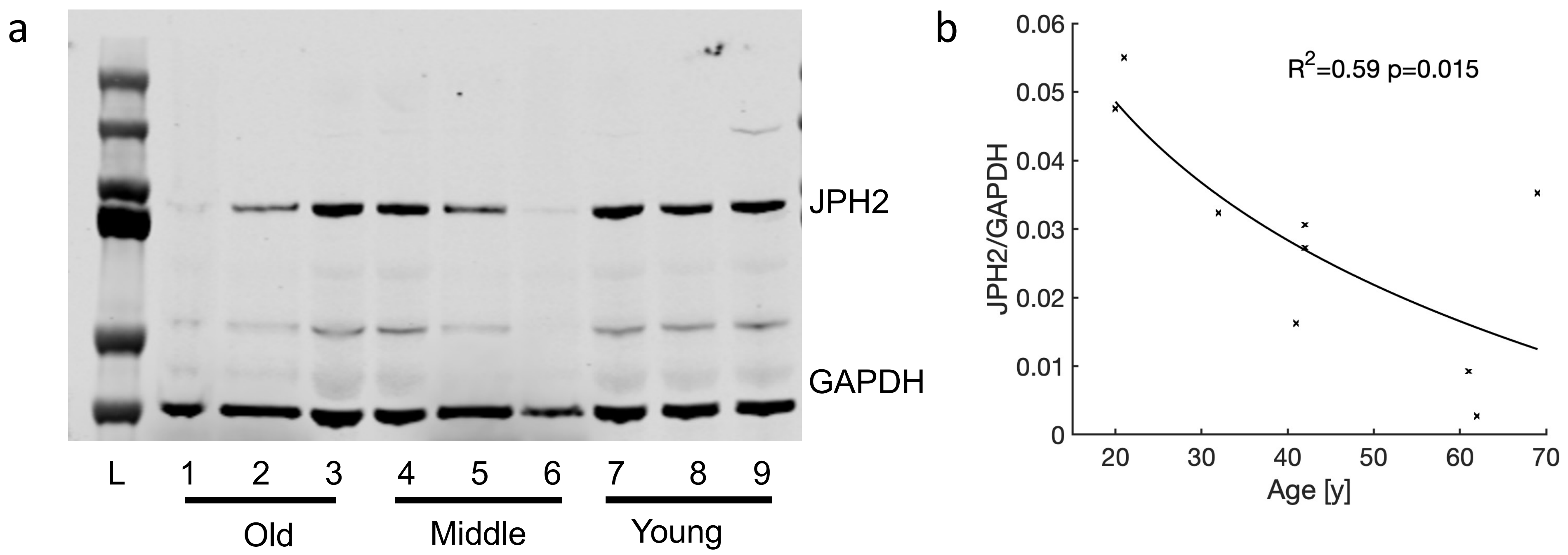

Supplementary Fig. 5: JPH2 expression in donor hearts. (a) Western blot of JPH2 and GAPDH. Lane marked L: Protein ladder, Lane 1-3: samples from old donors (age>55), Lane 4-6: samples from middle age donors (aged 35-55) and Lane 7-9: samples from young donors (age<35). (b) Quantification of JPH2. Increased age was associated with a decrease in JPH2 expression.

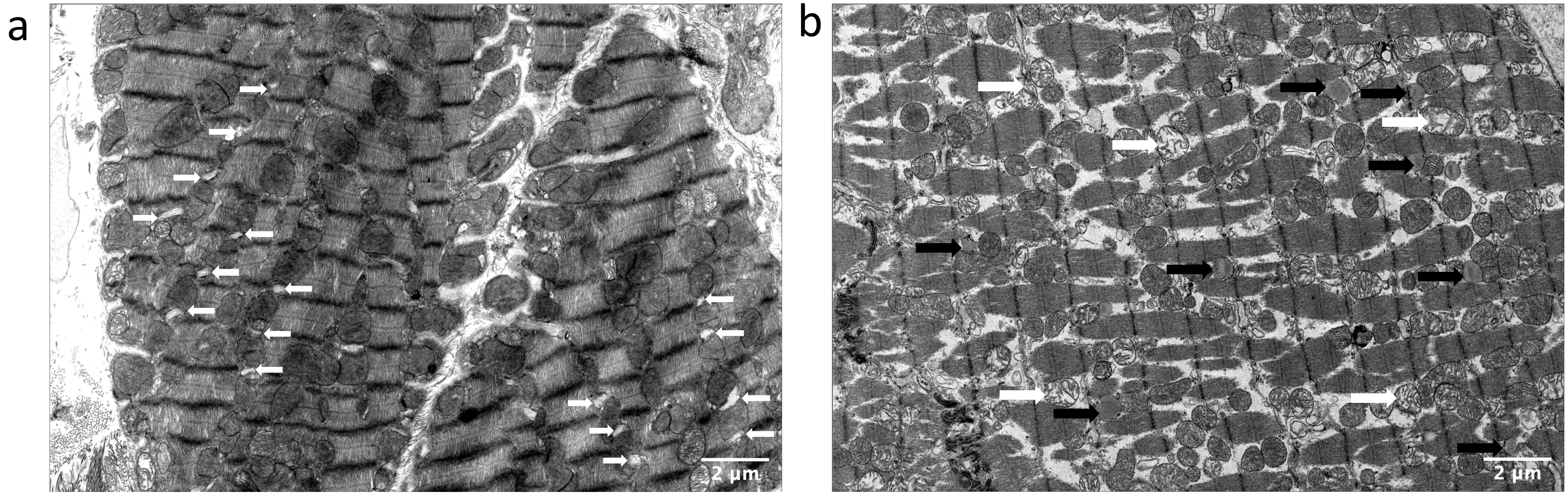

Supplementary Figure 6: Electron microscopy from LV tissue samples from young (31 yrs) and old donor (62 yrs). (A) The sample from the young donor exhibited densely packaged sarcomeres. The mitochondria were abundant. T-tubules (white arrows) were regionally dense and well aligned. (B) Old donor tissue exhibited a smaller density of sarcomeres. The tissue was also characterized by a less organized and reduced t-system. Mitochondria exhibited a reduced size and differed in the organization of cristae (white arrows). Furthermore, lipid droplets were observed in old donor tissue (black arrows).

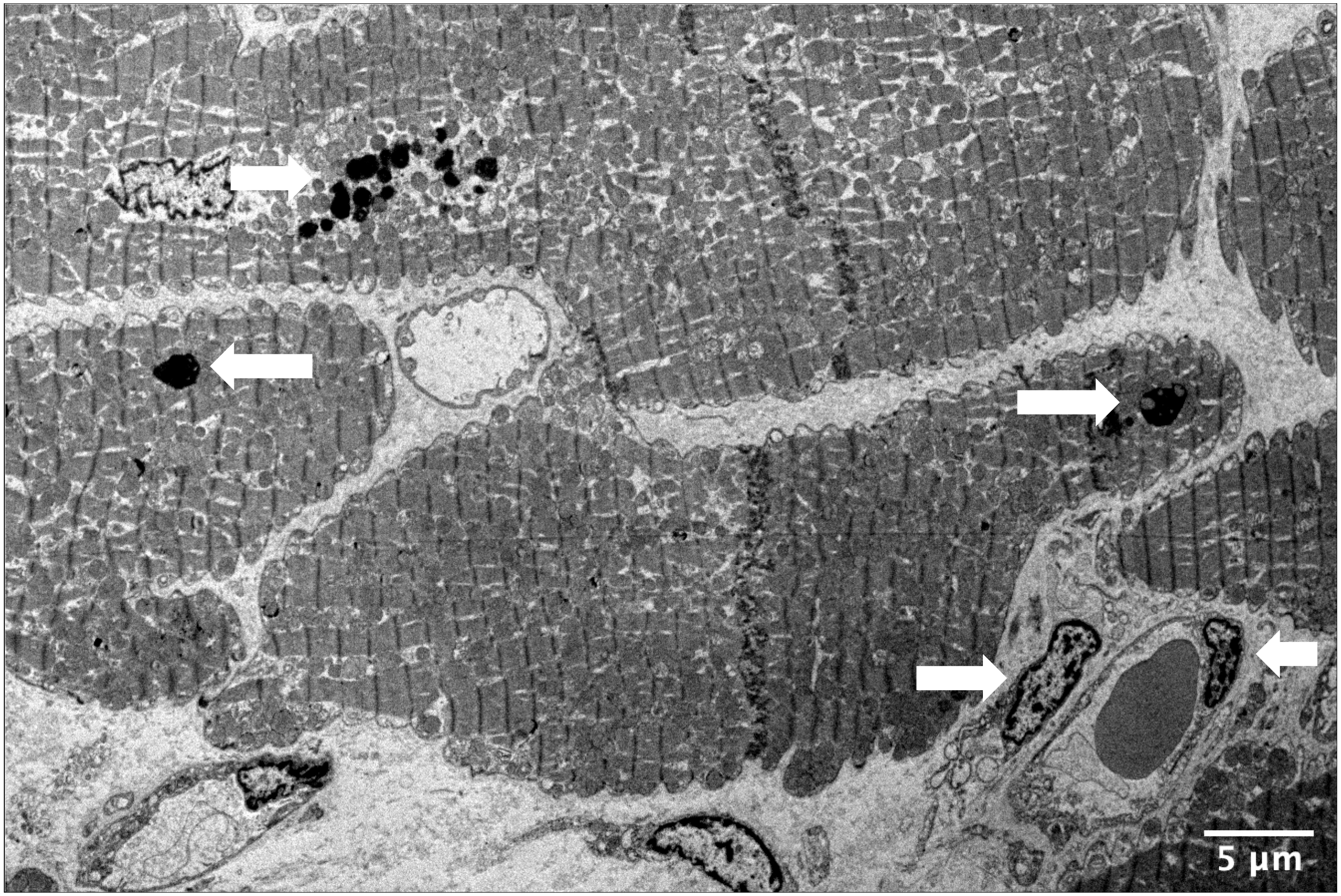

Supplementary Figure 7: Electron microscopy in LV tissue from old donor. Regions with high density (white arrow), presumably lipofuscin also known as age pigment, were frequently observed proximal to the nucleus of myocytes. Similar as in myocytes, regions with high density were present in other cells.

a b

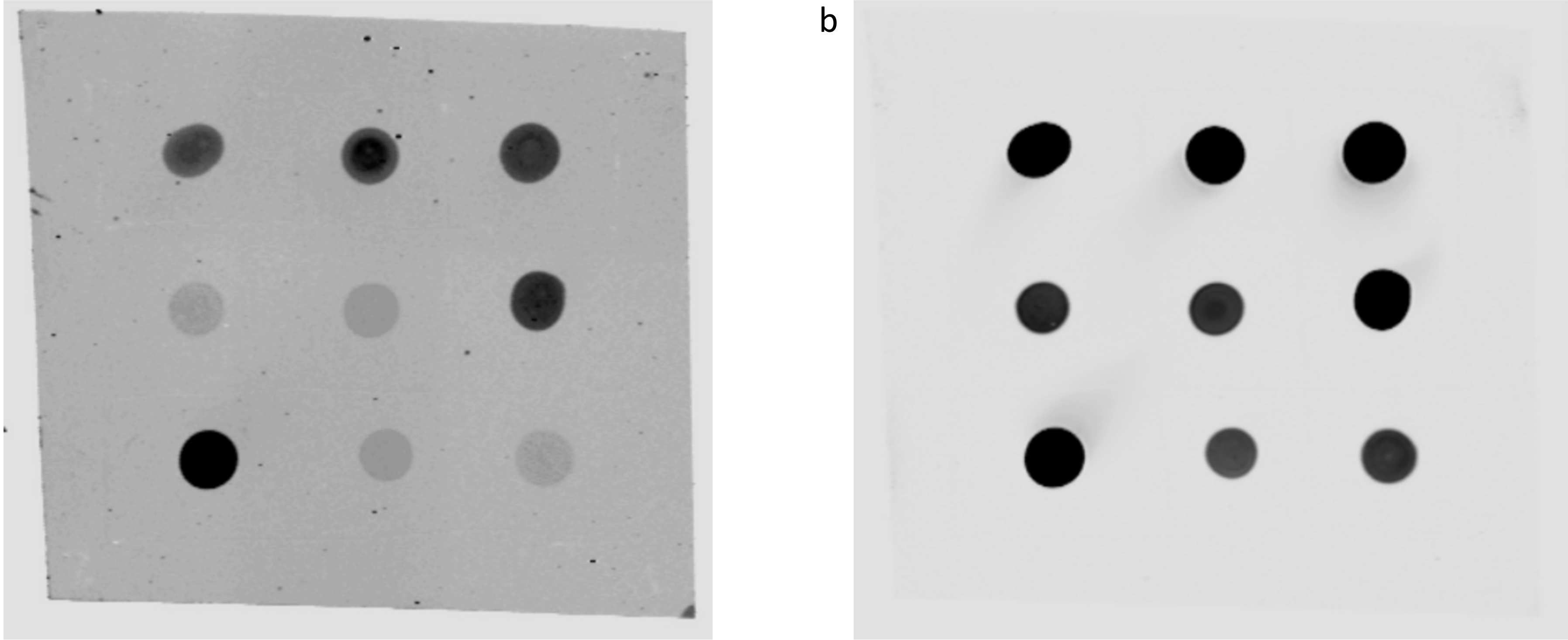

Supplementary Figure 8: Uncropped, full dot blot images for Supplementary Figure 4.

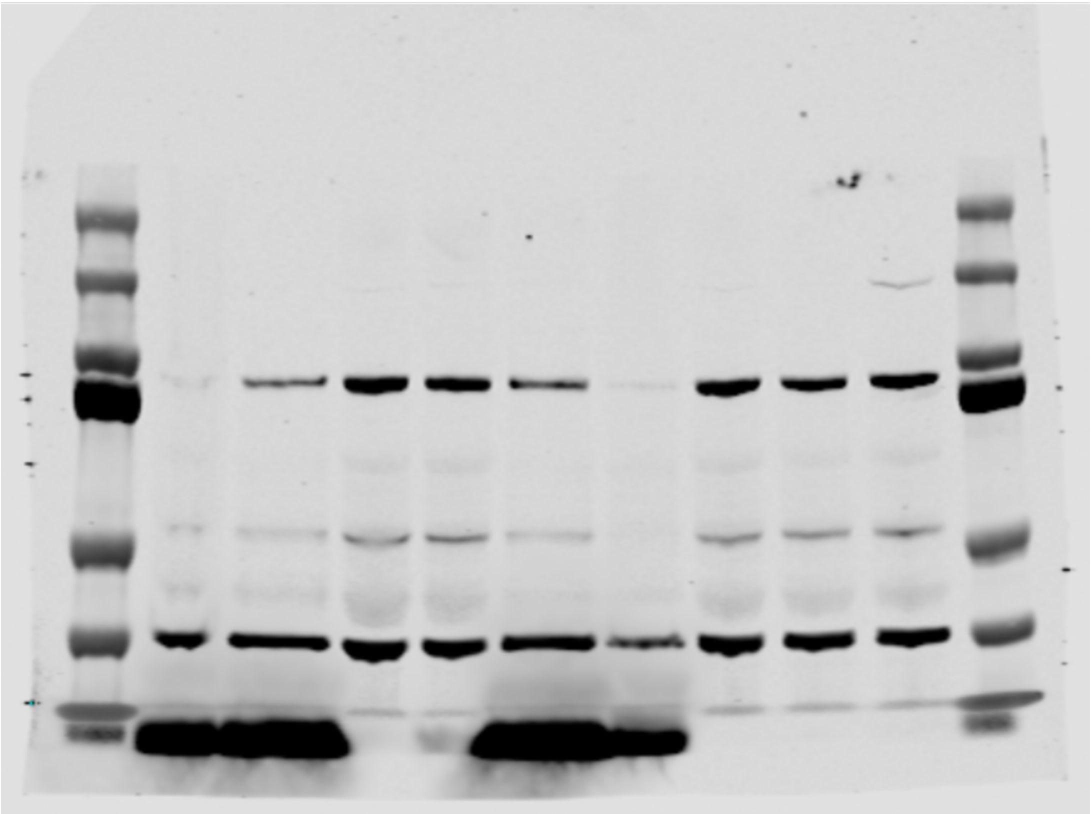

Supplementary Figure 9: Uncropped, full Western blot image for Supplementary Figure 5.
