# Supplementary material for: Remodeling of t-system and proteins underlying excitation-contraction coupling in aging versus failing human heart
Source: NPJ Aging Mech Dis. 2021 May 28;7:16. doi: 10.1038/s41514-021-00066-7 (PMC8163749; doi:10.1038/s41514-021-00066-7)
Supplement: Supplementary file 5 — Reporting Summary [file 41514_2021_66_MOESM5_ESM.pdf]

nature research

Corresponding author(s): Stavros Drakos, Frank Sachse

Last updated by author(s): 2021-5-3

Reporting Summary

Nature Research wishes to improve the reproducibility of the work that we publish. This form provides structure for consistency and transparency in reporting. For further information on Nature Research policies, see our Editorial Policies and the Editorial Policy Checklist.

Statistics

For all statistical analyses, confirm that the following items are present in the figure legend, table legend, main text, or Methods section.

n/a	Confirmed	
☐	☒	The exact sample size (*n*) for each experimental group/condition, given as a discrete number and unit of measurement
☐	☒	A statement on whether measurements were taken from distinct samples or whether the same sample was measured repeatedly
☐	☒	The statistical test(s) used AND whether they are one- or two-sided<br>*Only common tests should be described solely by name; describe more complex techniques in the Methods section.*
☒	☐	A description of all covariates tested
☒	☐	A description of any assumptions or corrections, such as tests of normality and adjustment for multiple comparisons
☒	☐	A full description of the statistical parameters including central tendency (e.g. means) or other basic estimates (e.g. regression coefficient) AND variation (e.g. standard deviation) or associated estimates of uncertainty (e.g. confidence intervals)
☐	☒	For null hypothesis testing, the test statistic (e.g. *F*, *t*, *r*) with confidence intervals, effect sizes, degrees of freedom and *P* value noted<br>*Give P values as exact values whenever suitable.*
☒	☐	For Bayesian analysis, information on the choice of priors and Markov chain Monte Carlo settings
☒	☐	For hierarchical and complex designs, identification of the appropriate level for tests and full reporting of outcomes
☐	☒	Estimates of effect sizes (e.g. Cohen's *d*, Pearson's *r*), indicating how they were calculated

Our web collection on statistics for biologists contains articles on many of the points above.

Software and code

Policy information about availability of computer code

Data collection	The data that support the findings of this study are available from the corresponding author upon reasonable request.
Data analysis	The code for processing and analyses in this study is available from the corresponding author upon reasonable request.

For manuscripts utilizing custom algorithms or software that are central to the research but not yet described in published literature, software must be made available to editors and reviewers. We strongly encourage code deposition in a community repository (e.g. GitHub). See the Nature Research guidelines for submitting code & software for further information.

Data

Policy information about availability of data

All manuscripts must include a data availability statement. This statement should provide the following information, where applicable:

- Accession codes, unique identifiers, or web links for publicly available datasets
- A list of figures that have associated raw data
- A description of any restrictions on data availability

The data that support the findings of this study are available from the corresponding author upon reasonable request.

Field-specific reporting

Life sciences study design

All studies must disclose on these points even when the disclosure is negative.

Sample size	Samples from the left ventricular free wall were obtained from 17 organ donors without a history of cardiac disease. Furthermore, 49 consented patients with advanced HF (New York Heart Association classes III or IV) were enrolled and provided tissue samples at the time of LVAD implantation (n=42) or heart transplantation without prior LVAD implantation (DTX, n=7).
Data exclusions	Images exhibiting weak signal-to-noise ratios or microstructural deterioration were excluded from the analyses.
Replication	Replication of the processing was assured by using Matlab scripts. Replication of the imaging was enhanced by providing pre-defined paramter setting and specific information on R0Is to the operator.
Randomization	Data were allocated to specific groups. Data from donor tissues were allocated to the donor group. Data from HF tissues were allocated to the HF group.
Blinding	All image processing and the figure generation for this study were implemented in Matlab scripts to assure reproducibility. The processing was applied in a consistent manner to all data.

Reporting for specific materials, systems and methods

We require information from authors about some types of materials, experimental systems and methods used in many studies. Here, indicate whether each material, system or method listed is relevant to your study. If you are not sure if a list item applies to your research, read the appropriate section before selecting a response.

Materials & experimental systems

n/a	Involved in the study	
☐	☒	Antibodies
☒	☐	Eukaryotic cell lines
☒	☐	Palaeontology and archaeology
☒	☐	Animals and other organisms
☐	☒	Human research participants
☐	☒	Clinical data
☒	☐	Dual use research of concern

Methods

n/a	Involved in the study	
☒	☐	ChIP-seq
☒	☐	Flow cytometry
☒	☐	MRI-based neuroimaging

Antibodies

Antibodies used	RyR2 monoclonal (MA3-916, Fisher Scientific), JPH2 polyclonal (40-5300, Fisher Scientific) , Alexa Fluor 633 goat anti-rabbit IgG (A21070, Invitrogen, MA), Alexa Fluor 488 goat anti-mouse IgG (A11001, Invitrogen) , GAPDH (5174, Cell Signaling Technology, Danvers, MA), GAPDH (ab37168, Abcam)
Validation	We have a long history of using the antibodies most important in this study. We assured that labeling is consistent with our prior work on various species.

Human research participants

Policy information about studies involving human research participants

Population characteristics	The donor and HF populations are extensively described in table 1 and 2, respectively.
Recruitment	Recruitment required informed consent of the HF patients. Gender and race were not considered for patient enrollment at the Utah Transplantation Affiliated Hospitals (U.T.A.H.) Cardiac Transplant Program.
Ethics oversight	IRB, University of Utah, Salt Lake City, Utah

Note that full information on the approval of the study protocol must also be provided in the manuscript.

Clinical data

Policy information about clinical studies

All manuscripts should comply with the ICMJEguidelines for publication of clinical research and a completedCONSORT checklist must be included with all submissions.

Clinical trial registration	All studies were authorized by the institutional review boards of the institutions comprising the Utah Transplantation Affiliated Hospitals (U.T.A.H.) Cardiac Transplant Program.

Study protocol	The study protocol is available through the IRB, University of Utah.
Data collection	Data collection was through the Utah Transplantation Affiliated Hospitals (U.T.A.H.) Cardiac Transplant Program. Data was only collected from patients after consent.
Outcomes	N/A
